# Identification and characterization of cherry (*Cerasus pseudocerasus* G. Don) genes responding to parthenocarpy induced by GA3 through transcriptome analysis

**DOI:** 10.1186/s12863-019-0746-8

**Published:** 2019-08-01

**Authors:** Binbin Wen, Wenliang Song, Mingyue Sun, Min Chen, Qin Mu, Xinhao Zhang, Qijie Wu, Xiude Chen, Dongsheng Gao, Hongyu Wu

**Affiliations:** 1 College of Horticulture Science and Engineering, Shandong Agricultural University, 61 Daizong Road, Tai’an, 271018 China; 2 State Key Laboratory of Crop Biology, Shandong Agricultural University, 61 Daizong Road, Tai’an, 271018 China; 3 Shandong Collaborative Innovation Center for Fruit and Vegetable Production with High Quality and Efficiency, Shandong Agricultural University, 61 Daizong Road, Tai’an, 271018 China

**Keywords:** Sweet cherry, GA3, Transcriptome, Parthenocarpy, Fruit set and cell division

## Abstract

**Background:**

Fruit set after successful pollination is key for the production of sweet cherries, and a low fruit-setting rate is the main problem in production of this crop. As gibberellin treatment can directly induce parthenogenesis and satisfy the hormone requirement during fruit growth and development, such treatment is an important strategy for improving the fruit-setting rate of sweet cherries. Previous studies have mainly focused on physiological aspects, such as fruit quality, fruit size, and anatomical structure, whereas the molecular mechanism remains clear.

**Results:**

In this study, we analyzed the transcriptome of ‘Meizao’ sweet cherry fruit treated with gibberellin during the anthesis and hard-core periods to identify genes associated with parthenocarpic fruit set. A total of 25,341 genes were identified at the anthesis and hard-core stages, 765 (681 upregulated, 84 downregulated) and 186 (141 upregulated, 45 downregulated) of which were significant differentially expressed genes (DEGs) at the anthesis and the hard-core stages after gibberellin 3 (GA3) treatment, respectively. Based on DEGs between the control and GA3 treatments, the GA3 response mainly involves parthenocarpic fruit set and cell division. Exogenous gibberellin stimulated sweet cherry fruit parthenocarpy and enlargement, as verified by qRT-PCR results of related genes as well as the parthenocarpic fruit set and fruit size. Based on our research and previous studies in *Arabidopsis thaliana*, we identified key genes associated with parthenocarpic fruit set and cell division. Interestingly, we observed patterns among sweet cherry fruit setting-related DEGs, especially those associated with hormone balance, cytoskeleton formation and cell wall modification.

**Conclusions:**

Overall, the result provides a possible molecular mechanism regulating parthenocarpic fruit set that will be important for basic research and industrial development of sweet cherries.

**Electronic supplementary material:**

The online version of this article (10.1186/s12863-019-0746-8) contains supplementary material, which is available to authorized users.

## Highlight

Cherry genes respond to parthenocarpy and promote fruit setting induced by GA3

## Background

Fruit set is an important step in fruit growth and development. In this process, the ovary becomes enlarged with development of the embryo after fertilization, inducing fruit formation [1]. A variety of hormonal synergies play a major role in controlling fruit set; GA, auxin, and cytokinin alone cause the fruit to grow and develop to a certain stage, but the fruit develops normally due to their synergistic effect [2, 3].

Exogenous spraying of 5 mg / L GA3 maintains the activity of citrus parietal cells and promote their division, thereby increasing the rate of fruit setting [4], and it was reported that treatment of grape flower spikes with 30 mg/L GA3 before full bloom significantly increased the fruit-setting rate [5]. Moreover, silencing of *SIGH3* and upregulation of *SIARF7*, a negative regulator of fruit set, in transgenic tomato plants decreased the IAA content; the number of middle and endocortical cells was also reduced, but their volume increased [6, 7]. In addition to auxin and gibberellin, the fruit-setting rate is affected by cytokinin, which influences fruit growth by promoting cell division [8]. Transcriptome analysis of normally pollinated and gibberellin-treated cucumbers showed that gibberellin promotes fruit set through interaction with auxin and cytokinin [9]. Therefore, knowledge of the transcriptome can help in an understanding of the molecular mechanism by which gibberellin regulates fruit set.

Parthenocarpy is an agronomic trait in which fruit develops without pollination and fertilization under natural conditions or with human intervention [10], and exogenous gibberellin can induce parthenocarpy during crop production. For example, application of 1500 mg / L GA3 during anthesis led to high-quality parthenogenetic fruit in *Annona lucidum* [11], and 500 mg / L GA_4 + 7_ induced parthenocarpy in sand pear [12]. Gibberellins are also active in inducing parthenogenesis and fruit development in other crops such as cucumber [13], lemon [4], tomato [14], and banana [15]. Overall, gibberellin affects plant growth and development, and there are two main genes that function in the regulation of GA signaling. The protein encoded by the *DELLA* gene represses gibberellin signaling; binding of gibberellins to the gibberellin receptor *GID1*, stimulates ubiquitination of the DELLA protein, resulting in 26S proteasome-mediated degradation. In *SIDELLA-*silenced transgenic tomato, gibberellin synthesis pathway-related gene expression is altered, and parthenocarpy is induced via promotion of ovarian cell division and growth [16–20].

Sweet cherry is a delicious and juicy fruit, the production of which has become a major industry, providing farmers with increasing income. However, the low fruit-setting rate caused by self-fruiting or its low self-setting rate has become a bottleneck restricting the development of this industry. As exogenous gibberellin can induce parthenocarpy in fruit, thereby increasing the fruit-setting rate, we sought to elucidate the molecular mechanism by which gibberellin induces parthenocarpic fruit set. To this end, we used next-generation sequencing technology to carry out transcriptome sequencing (RNA-seq) at two key developmental periods after GA3 treatment of ‘Meizao’ European sweet cherry grown under greenhouse conditions. This is the first application of RNA-seq to analyze developmental metabolic pathways and gene expression patterns in sweet cherry fruit after GA treatment. Genes that promote fruit setting, parthenogenesis, cell division and growth were among those analyzed. The aim of this study is to provide evidence for the molecular mechanism of fruit enlargement and fruit setting and to examine the effect of plant hormone regulation on the growth and development of fruit in parthenogenetic European sweet cherry treated with GA3. The differentially expressed genes identified through transcriptome sequencing provide a molecular foundation explaining the induction of parthenocarpic fruit set by exogenous gibberellin in sweet cherry.

## Results

### Growth and development characteristics of fruit after exogenous GA3 treatment

**Fig. 1:**
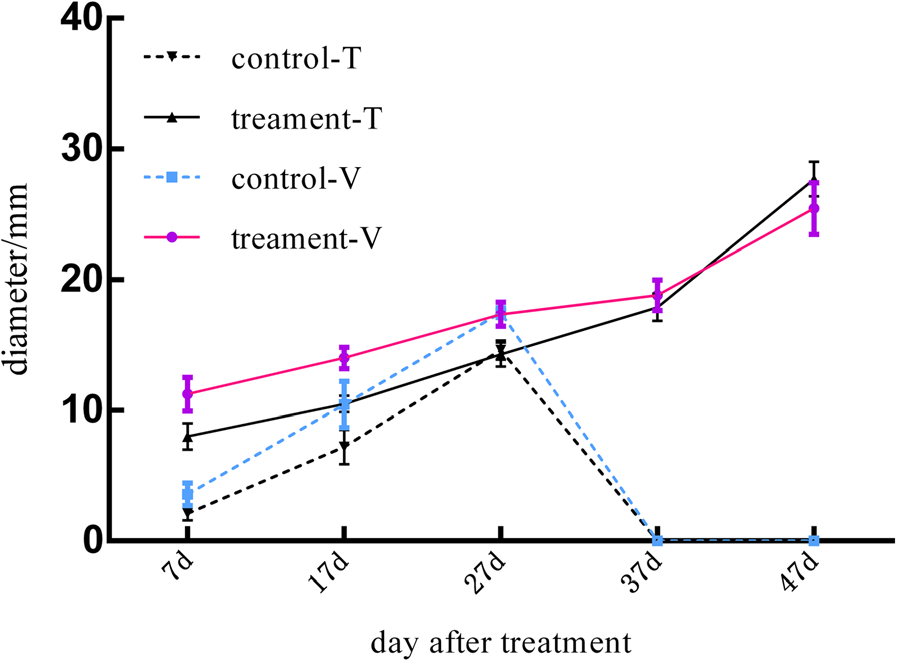
Growth curves of sweet cherry fruits in the treatment and control groups, respectively. T, transverse diameter; V, vertical diameter

Full growth and development of fruit was observed after treatment of ‘Meizao’ sweet cherry trees in the anthesis period (7 DAT) and the hard-core period (27 DAT) with 300 mg/L GA3. The size of GA3-treated sweet cherry is shown in Table 1. At 7 DAT, the vertical and transverse diameters of sweet cherry treated with GA3 were significantly higher than those of the control. Among them, the increase in the vertical diameter was higher than the increase in the transverse diameter. In the anthesis stage (7 DAT), the diameter of GA3-treated fruits increased by 123.9% compared with the control group, and the vertical diameter increased by 214.09% (Fig. 1). In the first expansion period (17 DAT), the growth rate of the transverse diameter was not significantly different from that of the control, but the vertical diameter reached 34.16%. After the hard-core stage (27 DAT), all the fruits of the control group had dropped.

**Table 1:** The size of GA3-treated sweet cherry

	Treatment	7 DAT	17 DAT	27 DAT	37 DAT	47 DAT
Transverse diameter/mm	CK1	3.577b ± 0.872	10.437a ± 1.766			
	T1	8.01e ± 1.009	10.488d ± 0.613	14.268c ± 0.922	17.895 ± 1.053	27.698a ± 1.328
Growth rate/%		123.9	0.49			
vertical diameter/mm	CK2	3.577b ± 0.872	10.437a ± 1.766			
	T2	11.235e ± 1.287	14.002d ± 0.805	17.359c ± 0.913	18.83b ± 1.165	25.434 ± 1.984
Growth rate/%		214.09	34.16			

Different lowercase letters indicate significant differences between the different treatments at the *p* < 0.05 level

*CK* Control groups without GA3 treatment

*T* GA3 treatment groups

*DAT* Days after treatment

*Growth rate* (GA3 treatment–control)/control× 100%

### Fruit setting rate and parthenogenetic rate of fruit after exogenous GA3 treatment

**Fig. 2:**
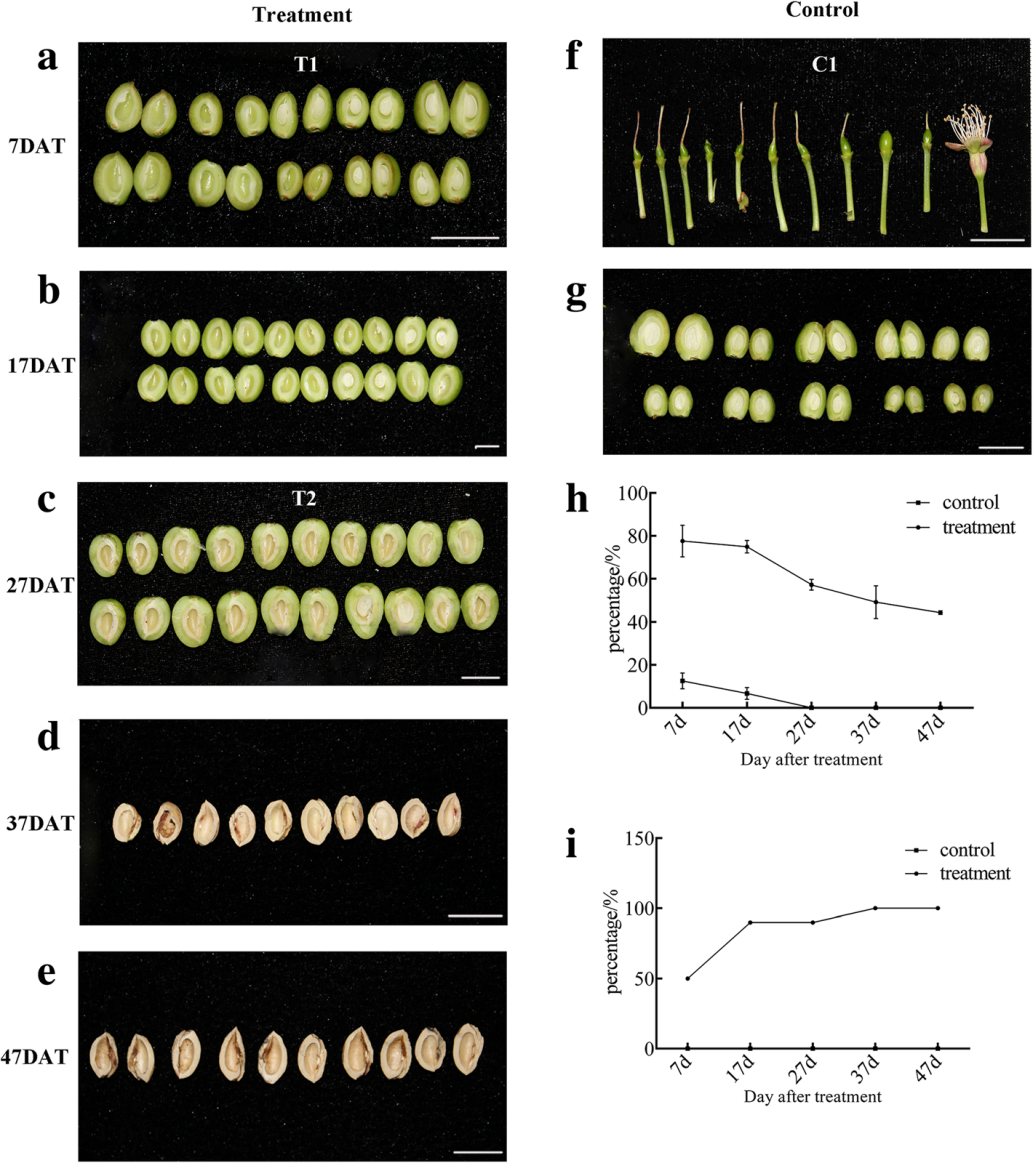
Effect of GA3 treatment on fruit shape, fruit-setting rate and parthenogenetic rate of sweet cherry. **a** Treatment: 7 days after treatment (7 DAT); **b** 17 DAT; **c** 27 DAT; **d** 37 DAT; **e** 47 DAT; **f** Control: 7 days after full bloom; **g** 17 days after full bloom; **h** Fruit-setting rate; **i** Parthenogenetic rate

After a week of spraying GA3 at the anthesis stage (7 DAT), the fruit set rate was 77.33% and the control 12.55%. During the hard-core period (27 DAT), the untreated sweet cherries had completely dropped, whereas the GA3-treated sweet cherries maintained a fruit setting rate of 57.23%. As shown in Fig. 2h, spraying GA3 in the blossom drop period can both induce parthenogenesis and significantly increase the fruit-setting rate. After GA3 treatment, the rate at which fruit dropped from the first expansion to the second enlargement was 34.3%, and the parthenogenetic rate was 100% (Fig. 2i). With the growth of the fruit, the development of the seeds was inhibited, and embryo abortion occurred.

### Analysis of transcriptional sequence data

Illumina platform sequencing revealed 137,658,266 (total of CK11, CK12, CK13) and 134,689,144 (total of CK21, CK22, CK23) and 140,680,232 (total of T11, T12, T13) and 138,579,108 (total of T21, T22, T23) raw reads for the control and GA-treatment groups, respectively (Table 2). After removing linkers, low-quality reads and reads with an N ratio greater than 5%, 136,440,870, 133,557,948, 139,421,700 and 137,336,514 clean reads were selected for further analysis. The proportion of clean reads to total reads obtained from the four libraries was 99.11% (average of CK11, CK12, CK13), 99.16% (average of CK21, CK22, CK23), 99.11% (average of T11, T12, T13) and 99.10% (average of T21, T22, T23). Among these reads, 83, 83.67, 83, and 79.67% were mapped to the European sweet cherry genome (https://www.rosaceae.org/species/prunus_avium/genome_v1.0.a1), including 81.65, 80.87, 80.86, and 81.22% mapped to exonic regions, 8.62, 9.45, 9.29, and 9.12% mapped to intronic regions, and 9.74, 9.69, 9.85, and 9.66% mapped to intergenic regions (Table 2).

**Table 2:** Quality control of the clean data

Sample	Raw of Number	Clean read%	Remove Adapter %	Ns Reads Rate %	Low quality %	Clean Q30 Bases Rate (%)	Mapping Rate %	Exon %	Intron %	Intergenic %
CK11	45,723,590	99.27	0.7	0.02	0	92.87	83	81.2	9.01	9.8
CK12	45,297,964	98.82	1.16	0.02	0	92.77	80	81.51	8.38	10.12
CK13	46,636,712	99.25	0.73	0.02	0	92.37	86	82.23	8.46	9.31
CK21	44,993,708	99.14	0.84	0.02	0	92.39	85	81.04	9.16	9.81
CK22	44,432,270	99.28	0.7	8.55E–05	0.02	91.63	84	80.06	10.15	9.79
CK23	45,263,166	99.06	0.91	0.02	0	92.17	82	81.5	9.04	9.46
T11	45,225,288	99.07	0.91	0.02	0	92.41	82	81.03	9.22	9.75
T12	47,694,260	98.96	1.02	0.02	0	92.13	86	82.08	8.45	9.47
T13	47,760,684	99.29	0.68	0	0.03	89.68	81	79.47	10.21	10.33
T21	45,251,876	99.11	0.87	0.02	0	92.46	76	81.01	9.35	9.64
T22	47,477,702	98.99	0.98	0.02	0	92.32	83	81	9.15	9.85
T23	45,849,530	99.21	0.77	0.02	0	92.38	80	81.66	8.86	9.48

The Ns reads rate indicates that N is too high; the number of sequences that have been removed accounts for the proportion of the original sequence number

Intergenic: new transcripts, expression noise, etc., result in sequences derived from intergenic regions

*CK1* control groups without GA treatment in the anthesis stage

*CK2* control groups without GA treatment in the hard nucleus stage

*T1* GA treatment groups in the anthesis stage

*T2* GA treatment groups in the hard-nucleus stage

### Identification and classification of DEGs at different stages

**Fig. 3:**
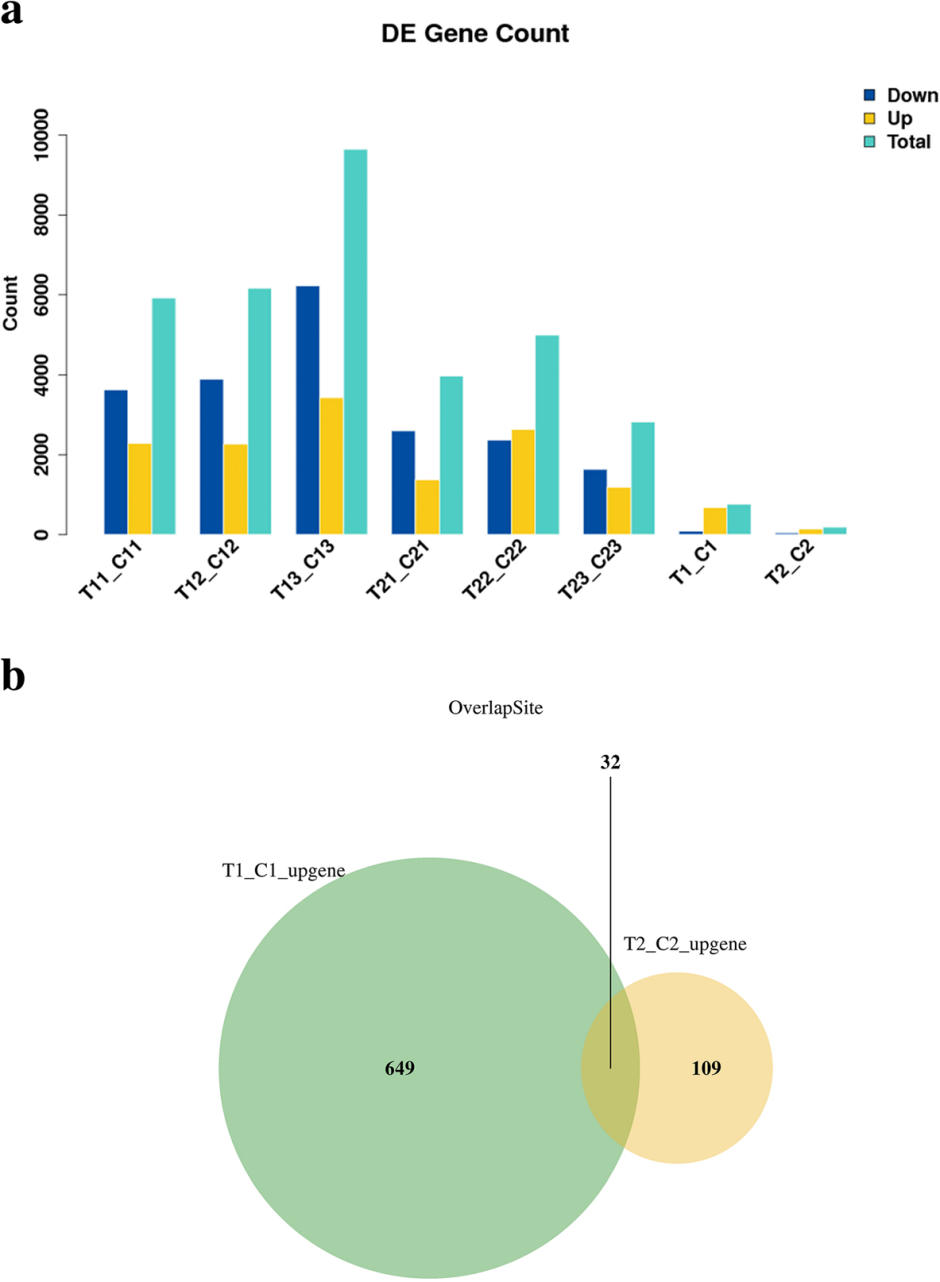
Analysis of DEGs at the two key sweet cherry developmental stages (anthesis and hard-core stages). **a** Numbers of DEGs between the control and GA3-treated samples at the two developmental stages; **b** Venn diagram of the up-regulated genes identified at the two sweet cherry development stages

In the two key developmental stages of European sweet cherry, changes in gene expression were determined by comparing C1 versus T1 and C2 versus T2. In these two periods, 765 and 186 significant differentially expressed genes were obtained. In the first period, 685 genes were upregulated, and 84 genes were downregulated. In the second period, 141 upregulated and 45 downregulated genes were identified by RNA-seq (Fig. 3a). The number of upregulated genes was significantly higher than that of downregulated genes in the two periods of European sweet cherry development. However, Venn diagram analysis indicated that only 32 genes showed a pattern of upregulated expression in both key periods (Fig. 3b), and specific upregulated genes were detected at every time point.

### Changes in expression patterns of genes related to gibberellin metabolism and signal transduction

After treatment with gibberellin, the fruit rate, fruit size and rate of parthenocarpy changed significantly. Gibberellin 2-beta-dioxygenase (*GA2ox*) is a key enzyme of GA catabolism [21], and the level of *GA2ox* expression changed significantly during anthesis; it was also detected during the hard-core period. *Pav_sc0000095.1_g1110.1.mk* was significantly upregulated at 7 DAT (Additional file 2); the annotation is *PavGA2ox*, which was used to generate GA51, GA29, GA34 and GA8 in GA9, GA20, GA4 and GA1, respectively [22]. In *Arabidopsis thaliana*, the proteins *Scarecrow-like* (*SCL*) and *DELLA* are antagonists that promote gibberellin signaling [23]. Transcriptome sequencing revealed certain genes at the anthesis stage after GA treatment, such as *pavscl1, pavscl3* and *GA2ox*. Encoding a GA receptor, *GID1B* (*Pav_sc0000848.1_g080.1.mk*), was downregulated after GA3 treatment in the hard-nucleus phase, whereas *GA2ox* and *DELLA* were upregulated. These results showed that exogenous GA3 is associated with parthenogenesis.

In GA signal transduction, active GA regulates the hydrolysis and inactivation of *DELLA* by stimulating the *GID1-DELLA-GID2* complex, thus regulating downstream gene expression [24]. In this study, we found that the gene encoding *GID1* was downregulated after GA treatment and that expression of the *DELLA* gene was upregulated; however, the gene encoding *GID2* was not detected. *Pav_sc0000848.1_g080.1.mk* was further annotated as *PavGID1B* and only noted in the hard-core period. During the hard-core stage, the *DELLA* protein *Pav_sc0000464.1_g350.1.mk* significantly increased. The above results indicate that after treatment of ‘Meizao’ sweet cherry with exogenous GA3, no significant expression of *GID1-DELLA-GID2*-related genes was detected at the full anthesis stage. We speculate that *GID1-DELLA-GID2-*related genes respond very quickly and are correlated with seed development.

### Changes in expression patterns of cytoskeleton and cell wall modification genes

After GA3 treatment, significant changes in the size and shape of ‘Meizao’ cherries were noted. After 7 days, the treated fruits were significantly swollen (Fig. 2). Upregulation of plant hormone synthesis, degradation, control and signal transduction genes and downregulation of genes involved in the cytoskeleton, cell wall modifications and other related biological processes synergistically caused irreversible fruit expansion (Additional file 3). Expression of genes encoding actin and tubulin changed significantly after GA3 treatment. At 7 DAT, only one gene encoding actin, *EDH1* (*Pav_sc0001084.1_g180.1.mk*), was downregulated. Conversely, genes encoding tubulin were not detected during the hard-core period. In addition, different genes involved in cell division, including a cyclin-dependent kinase and five histones, were downregulated at the anthesis stage after GA treatment.

Expression of cell wall modification genes was significantly altered after GA treatment. Expansion proteins catalyze the extension of cell walls [25]. In our study, 26 cell wall-associated DEGs were detected at the anthesis stage after GA treatment, and 5 cell wall-associated DEGs were detected during the hard-core period, including those encoding expansin, xyloglucan glycosyltransferase and glucanase; most of these genes (27/31) were upregulated. Clearly, changes in expression of genes associated with cell division and cell wall metabolism occur during fruit development.

### DEG analysis of transcription factors

**Fig. 4:**
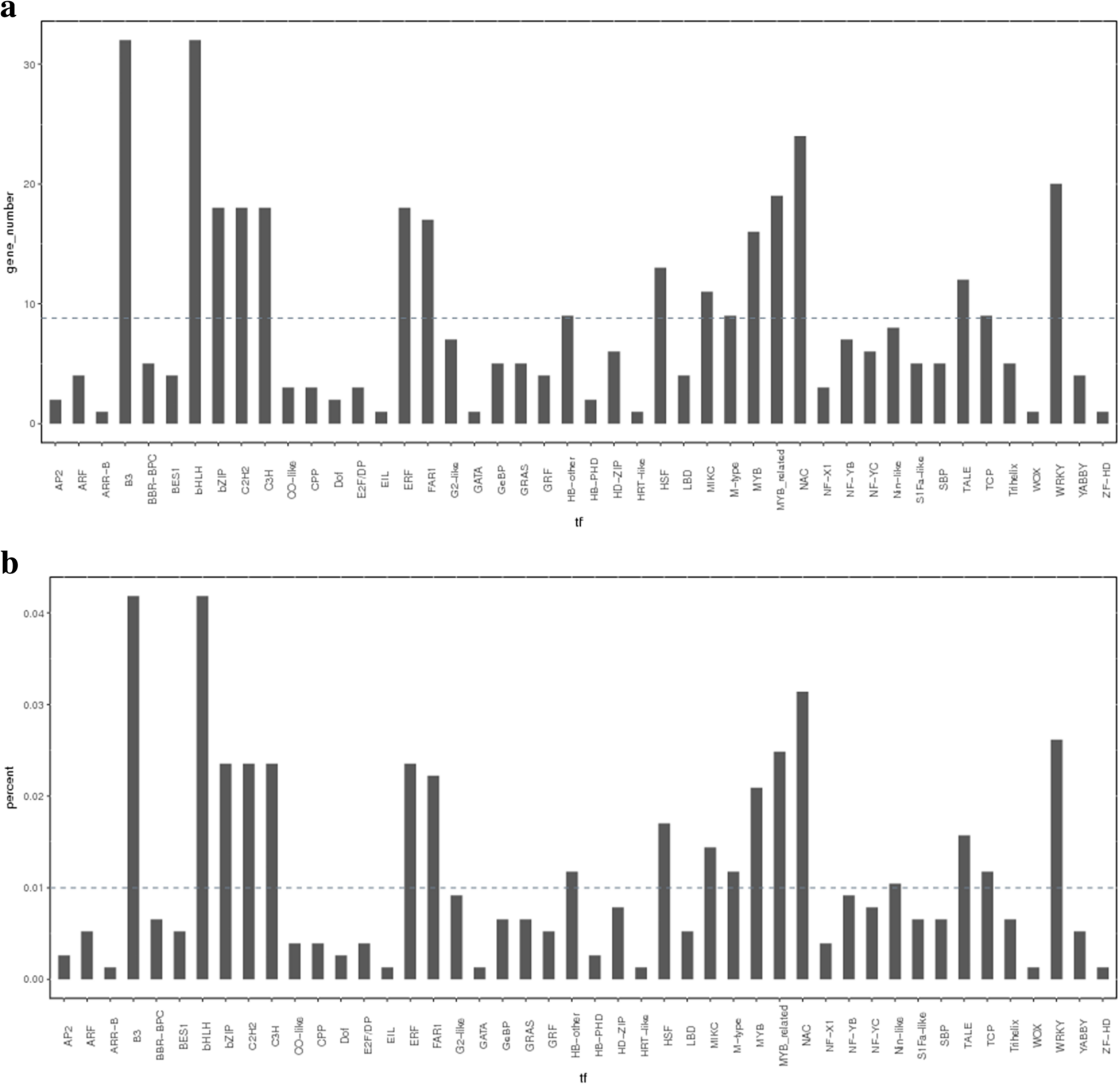
Analysis of DEGs in different transcription factor families. **a** The dashed line indicates the average number of DEGs for the different transcription factor families; **b** The proportion of different transcription factor families between the control and treatment groups relative to the total number of transcription factor families

**Fig. 5:**
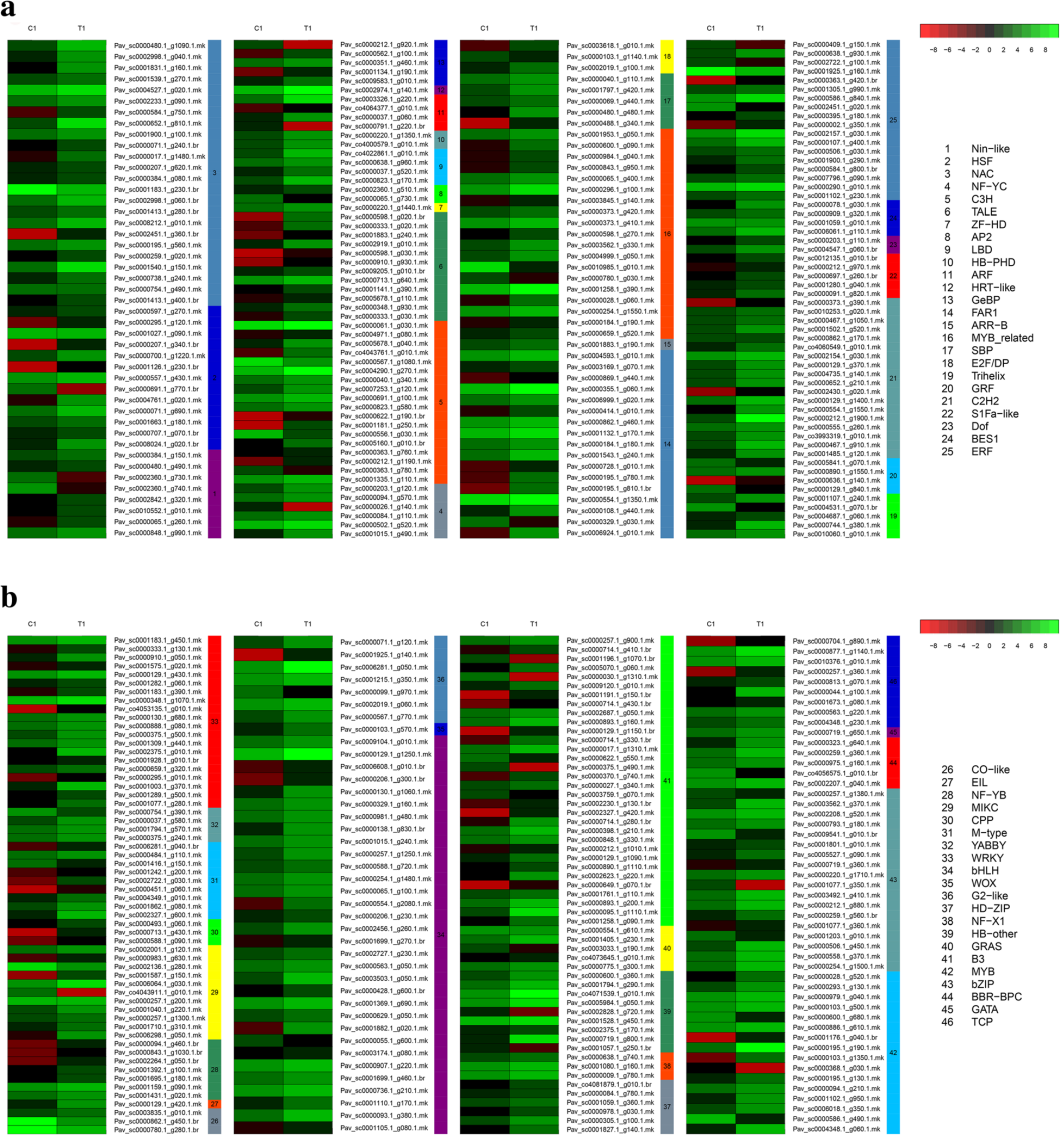
Expression analysis of genes from different transcription factor families (T1/C1)

**Fig. 6:**
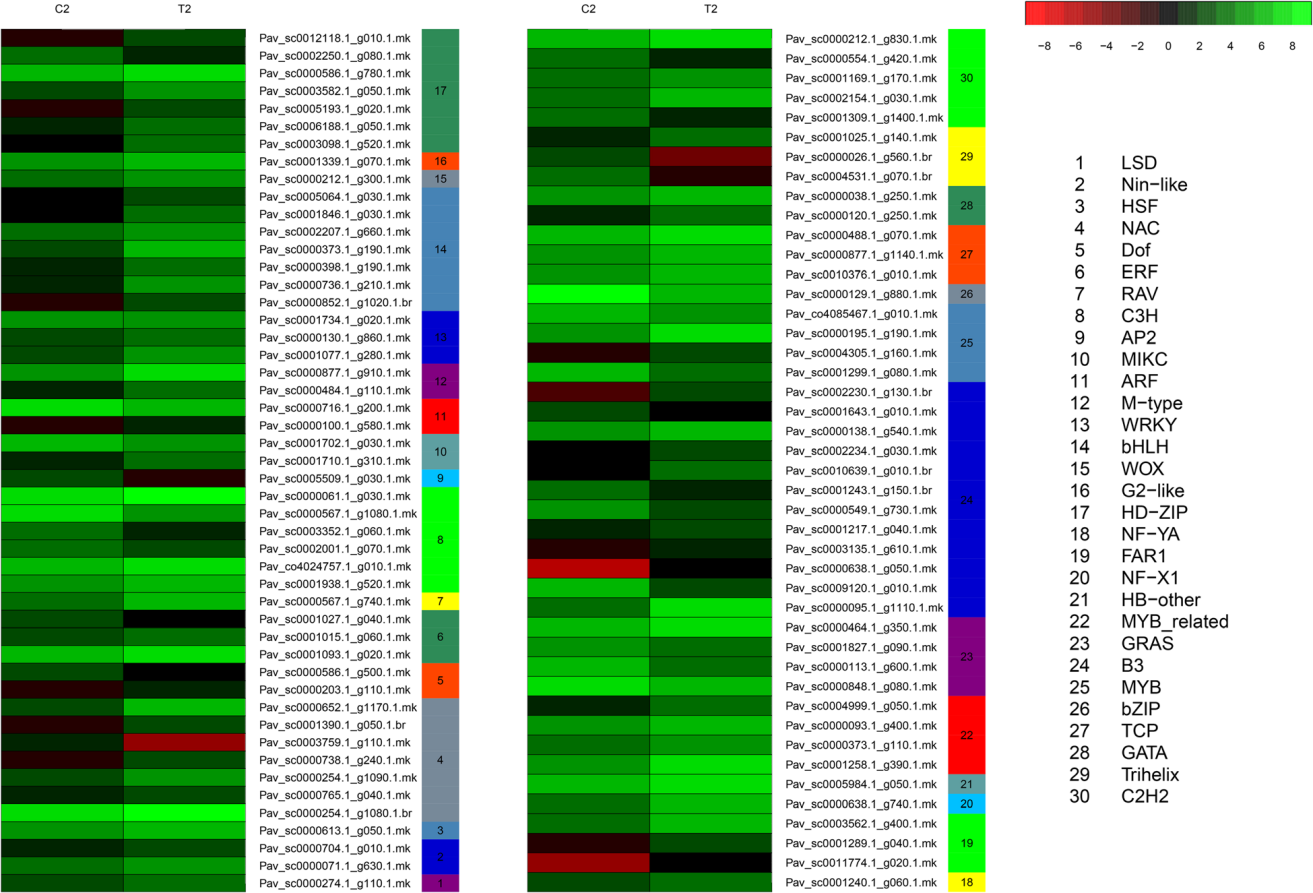
Expression analysis of genes from different transcription factor families (T2/C2)

Analysis of all DEGs at the two stages uncovered 403 transcription factors, which were further divided into 46 and 30 gene families (Fig. 4a). At anthesis, the transcription factor families with the most DEGs were the bHLH and B3 families (7.94%), followed by the NAC family (5.96%), the WRKY family (4.96%), the MYB-related family (4.71%), the C3H, C2H2, ERF and bZIP families (4.47%), the FAR1 family (4.22%), the MYB family (3.97%), the HSF family (3.23%), the TALE family (2.98%), the ARF family (2.73%), and the M-type, HB-other, and TCP families (2.23%). DEGs were strongly enriched in these 17 families of transcription factors. In the hard-core stage, the B3 family of transcription factors had the most DEGs (12.9%), followed by the NAC, bHLH and HD-ZIP families (7.52%), the C3H family (6.45%), the C2H2 family (5.38%), and the MYB-related, GRAS and MYB families (4.3%) (Fig. 4b). The abovementioned families of transcription factors contained a higher than average number of DEGs. Based on the expression patterns of the DEGs identified, most DEG transcription factors exhibited a trend of upregulation compared with the control at the two key stages after gibberellin treatment. Although some of the DEGs belonging to the Nin-like, HSF, ARF, B3, HB-other, MYB, Bzip, GeBP, C3H and NF-YC families were downregulated after the first treatment, the NAC and Trihelix families were downregulated after the second treatment. As previous studies have shown, many transcription factors play an important role in seed development in a wide variety of plants [26]. For example, the B3 superfamily is key in seed development [27–29]. Moreover, all NAC genes are reportedly involved in the synthesis of cell secondary metabolites [30], and transcription factors (TFs) such as *GRAS* and *HB* participate in ABA and GA signaling [31] (Figs. 5, 6). In our study, transcription factors related to seed development were differentially expressed between seeded and parthenogenetic progeny, indicating that they are associated with parthenogenetic phenotypes.

### Analysis of DEG pathway enrichment to reveal possible pathways influencing parthenogenesis

**Fig. 7:**
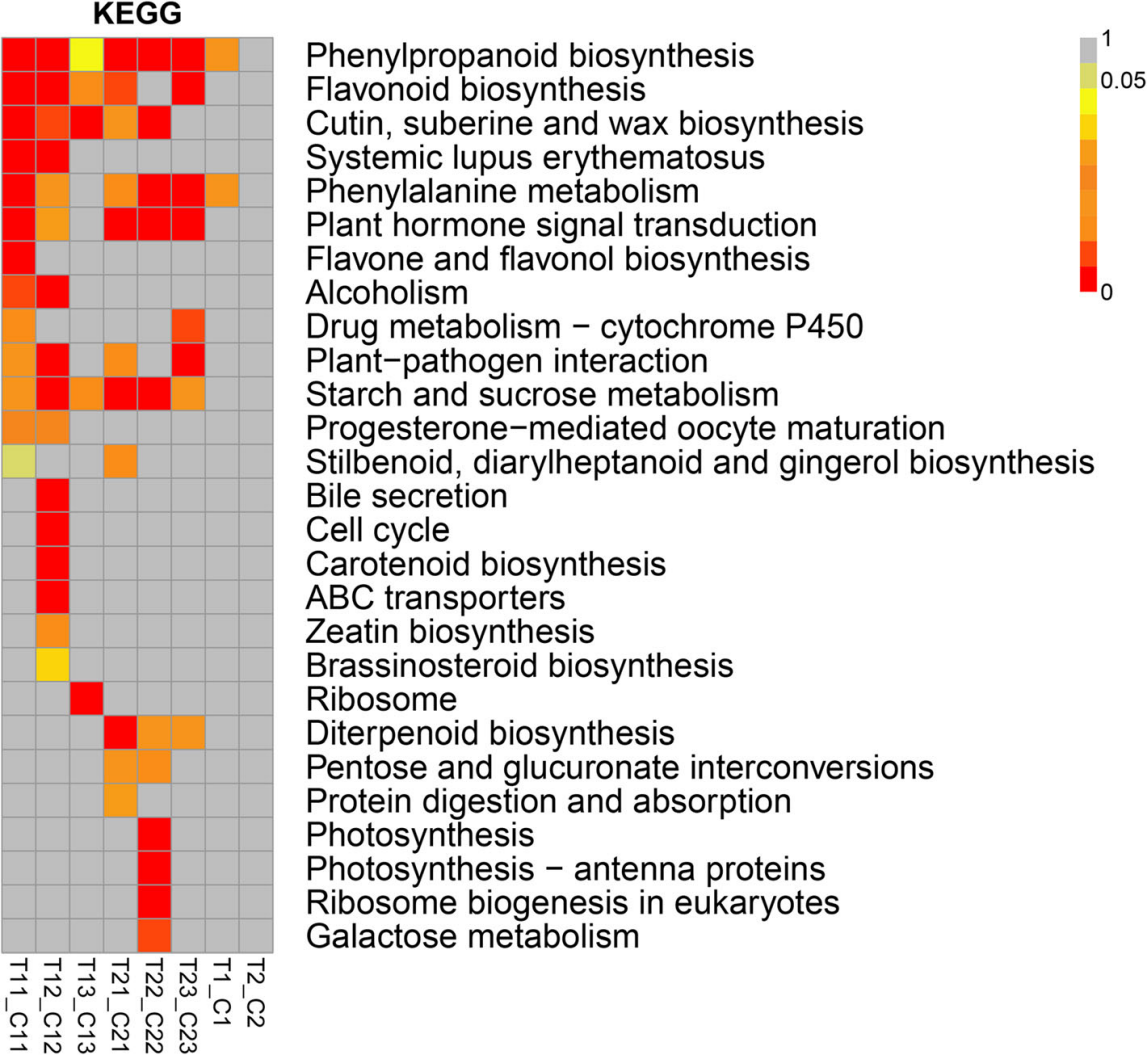
KEGG pathway analysis of DEGs at the two different developmental stages of sweet cherry

To further understand differences in the metabolic pathways of ovule development between seeded and parthenogenetic sweet cherry, we analyzed metabolic pathways showing differential gene enrichment in the two stages (Fig. 7), revealing 27 metabolic pathways enriched during the anthesis and hard-core periods. These 27 enriched metabolic pathways are involved in a variety of biological processes, including the synthesis and metabolism of secondary metabolites, plant hormone signal transduction, flavonoid biosynthesis, starch and sucrose metabolism, Cutin, and suberine and wax biosynthesis. Although no pathways were significantly enriched at the second developmental stage, two metabolic pathways, phenylalanine metabolism and phenylpropanoid biosynthesis, were significantly enriched at the first stage of cherry development, and most DEGs in these two pathways were upregulated. Collectively, these results suggest that parthenogenetic fruit set may be related to phenylalanine metabolism and phenylpropanoid biosynthesis pathways.

### Functional analysis of GOs of differential genes indicates factors that may lead to parthenogenetic fruit set

**Fig. 8:**
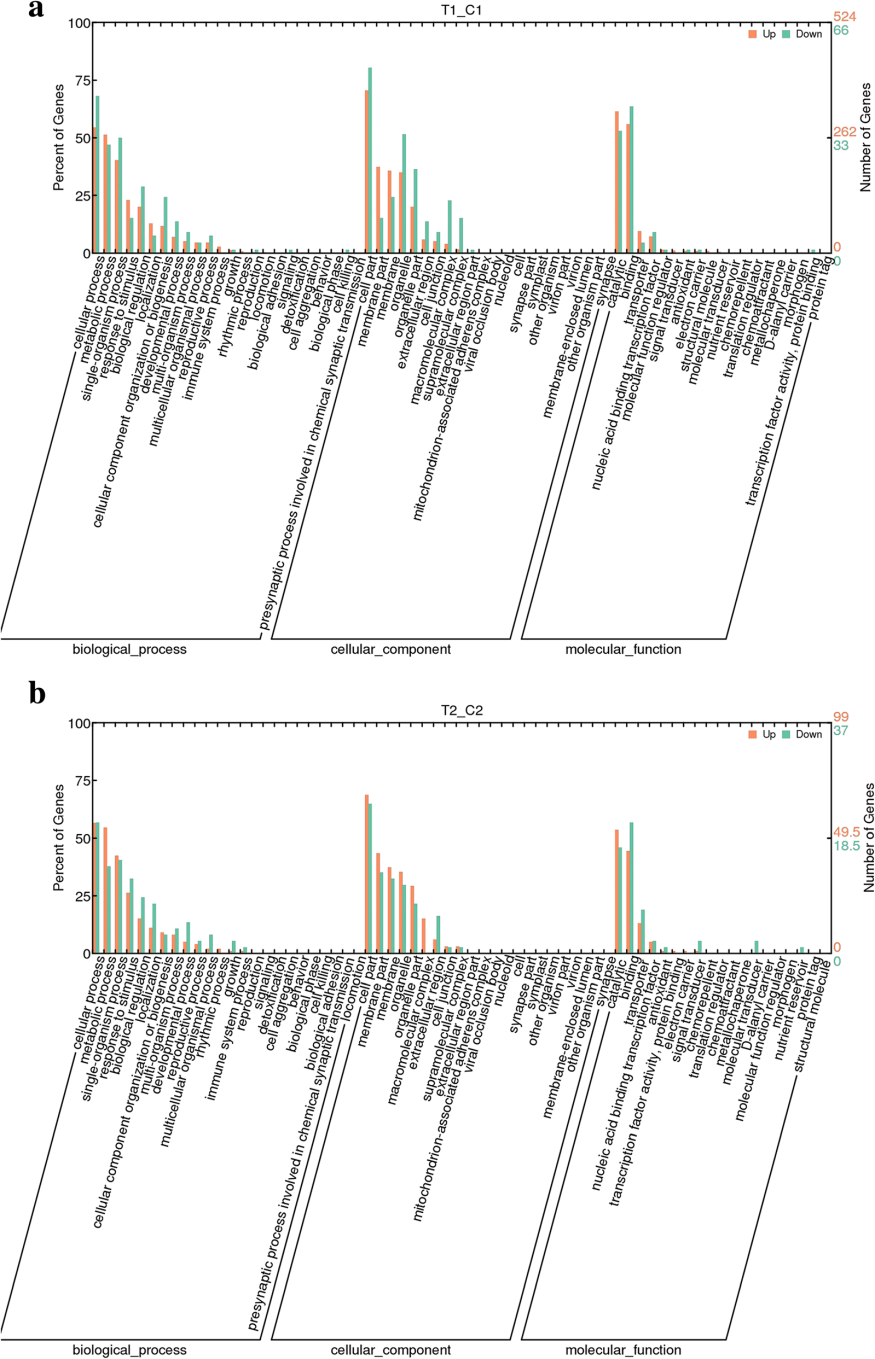
Gene ontology (GO) (corrected *P-*value≤0.05) analysis of DEGs between the control and GA3 treatments. **a** GO analysis of DEGs between the T1/C1 groups; **b** GO analysis of DEGs between the T2/C2 groups. The Y-axis represents the percentage of annotated DEGs for each GO term relative to the total number of DEGs

To explore the key biological processes and functional classification related to cherry parthenogenetic fruit set, DEGs were further analyzed for biological function GOs in the two critical stages of cherry development (Fig. 8). The biological functional classification of GOs involves the following three categories: biological process, molecular function and cellular component. Most of the DEGs were upregulated, and those detected at the first stage were highly represented in cellular process, metabolic process, single-organism process, response to stimulus, biological regulation, and cellular component organization of biogenesis groups in the biological process category. Most of the differences in gene-rich GOs were related to plant growth and development, response to external stimuli and signal transduction, glucose and lipid metabolism, and epigenetic regulation; the abovementioned groups and parthenogenetic fruit set are closely related. The status of differential gene enrichment was generally the same in the anthesis stage after GA treatment.

Based on the classification of GO-based molecular functions, DEGs were enriched in catalytic, binding, transporter and nucleic acid-binding transcription factor categories. These enriched GOs are closely related to the regulation of biological processes and anabolism of functional substances. In each subcategory, more genes were upregulated than downregulated in the first stage of sweet cherry development.

Further analyses of the GO classification of cellular components were performed, showing most DEGs to be enriched in the following groups: intrinsic component of membrane, integral component of membrane, membrane and membrane part. Similar to the GO enrichment of molecular function and biological process, in the GO enrichment of the cell group, most DEGs were upregulated in the anthesis stage after GA treatment compared with the control.

To further explore differences in the GO functions enriched among DEGs during the two critical stages of sweet cherry development, we focused on GO enrichment in the biological process category. During the two critical stages of sweet cherry development, 709/24 upregulated and 55/0 downregulated genes were found to be significantly enriched compared to the control. The GO items enriched in these two developmental stages are summarized below. In the anthesis stage, GO items with upregulated DEGs are mainly associated with cell wall development, with further division into the groups of cell wall synthesis and cytokinesis, carbohydrate and polysaccharide biosynthesis, secondary metabolism, and lignin and phenylpropanoid anabolism. During the anthesis stage, downregulation of GO items related to cell wall development was associated with DEGs significantly enriched in the cell cycle process, and GO items with upregulated DEGs are mainly associated with photosynthesis and sucrose biosynthesis. As indicated above, no downregulated genes were enriched in the second stage.

### Transcriptional dynamics analysis of DEGs

**Fig. 9:**
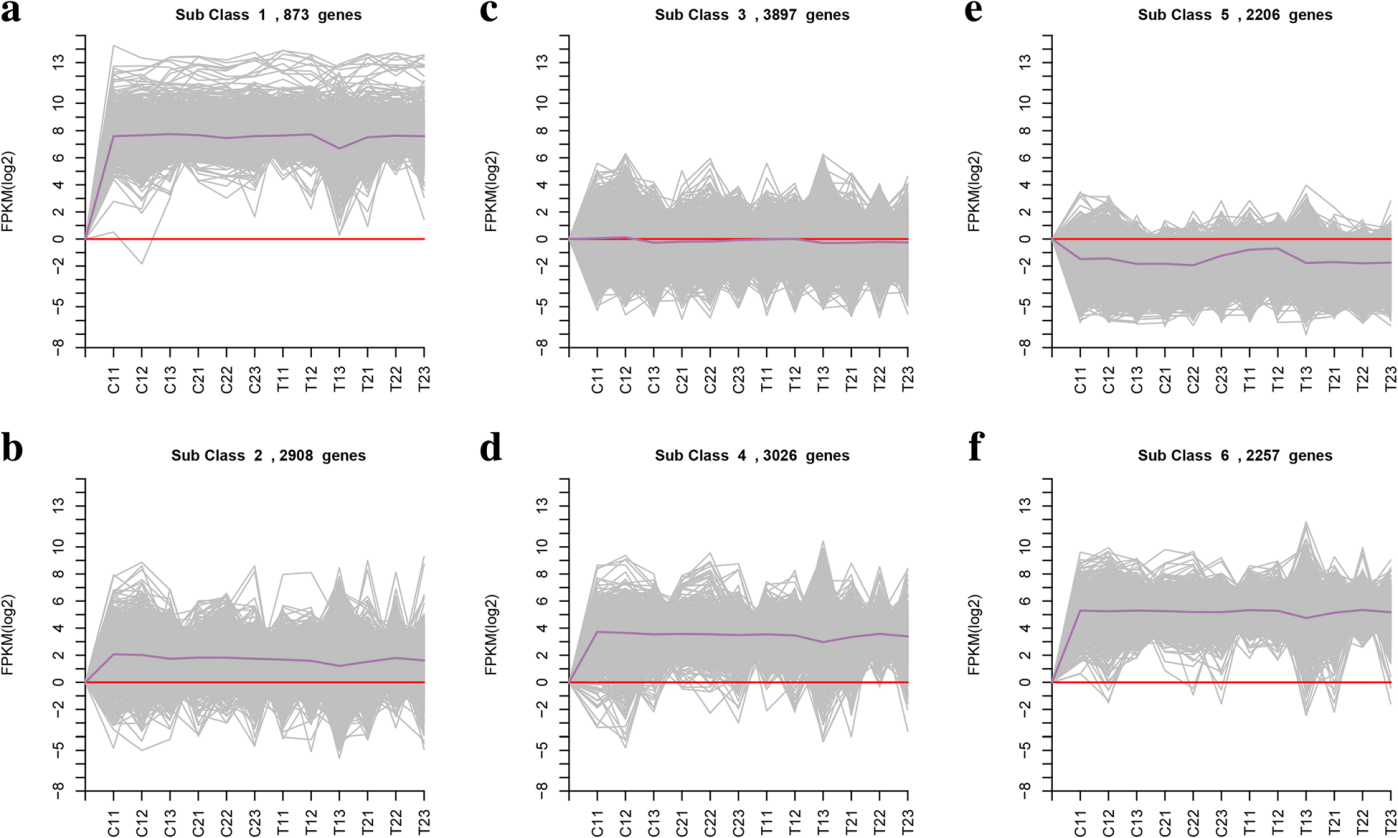
Expression pattern based on DEG clustering at the two key sweet cherry developmental stages. DEG clustering was performed using the k-means method. The number behind each cluster name represents the number of different genes in the cluster. The X-axis shows the different samples. The Y-axis shows the log_2_ RPKM values during the development of sweet cherry. The gray line indicates the gene expression changes between samples. The dark purple line represents the mean gene expression values. The red line indicates that the FPKM value (log_2_) is 0

We conducted further analysis of genes associated with fruit set, cell division and parthenocarpy in the anthesis and hard-core stages of sweet cherry using k-means clustering to classify genes with relatively consistent expression. The different genes (15,167 in total) were subjected to this analysis of temporal and spatial expression patterns, with all differentially expressed genes with similar expression patterns during the process of cherry development grouped into one group, as presented in Fig. 9. Six coexpressed clusters in the CK and GA3 treatments were identified during sweet cherry development, of which 4 were consistent in expression pattern. For parthenogenetic sweet cherry, DEGs were consistently upregulated in CK at the early stage of development in cluster 1, cluster 2, cluster 4, and cluster 6, and the only difference was the degree of upregulation. These four clusters displayed lower expression in the anthesis period after GA treatment. Cluster 5 exhibited different expression patterns, and the transcriptional level of the DEGs showed continuous downregulation. After GA3 treatment, the DEG patterns of cluster 5 and clusters 1, 2, 4, and 6 were reversed and correlated over time. The level of transcription was increased or decreased during anthesis in sweet cherry, and the differential genes between the five clusters exhibited completely opposite spatiotemporal expression patterns in the CK and GA3 treatments.

Based on clustering results, 5 of the transcription factors were selected for analysis, and it was found that the greatest numbers of transcription factors in cluster 5 belong to the AP2 and MYB families, followed by the MADS family. In cluster 5, the different genes are mainly enriched in GO items of seed and embryo development, cell differentiation, cell wall formation, defense response, hormone regulation, and transcriptional regulation, among others.

### RNA-seq validation of DEGs by quantitative real-time PCR (qRT-PCR)

**Fig. 10:**
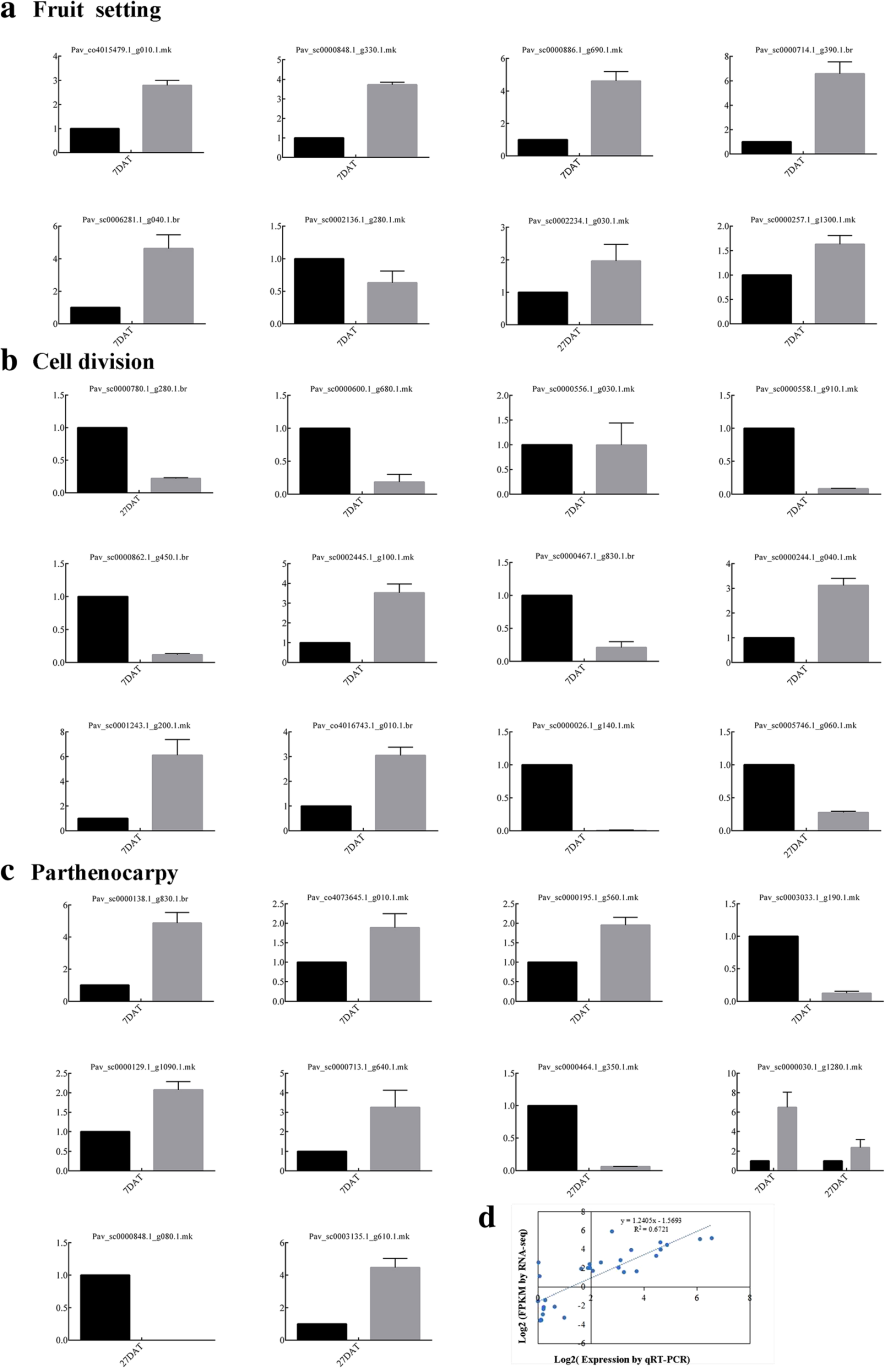
Expression analysis of genes participating in three biological processes. **a** Eight genes related to fruit setting were selected for qRT-PCR analysis; **b** Twelve genes related to cell division were selected for qRT-PCR analysis; **c** Ten genes related to parthenocarpy were selected for qRT-PCR analysis; **d** Coefficient analysis between the qRT-PCR and RNA-seq results

To validate the reliability of the transcriptome data, we applied qRT-PCR to detect expression of 30 DEGs related to fruit set, cell division and parthenocarpy in samples from the treatment and control groups (Fig. 10) and further carried out correlation analysis of gene expression fold changes. As shown in Fig. 10d, the results were highly consistent and thus verified the reliability of the transcriptome sequencing data. These 30 genes are mainly involved in the following processes: fruit setting (Fig. 10a), including *PavYUCCA*, *PavYUCCA10, PavACS, PavACO* and *PavCYP707A3*; cell division (Fig. 10b), including *PavH4, PavCDC2D, PavH3.2,* and *PavCDC45*; parthenocarpy (Fig. 10c), including *PavSAUR32*, *PavSCL1*, *PavCYCPA3*, and *PavDELLA*. The qRT-PCR results were consistent with the RNA-seq data, which strongly increased the reliability of our data.

## Discussion

Transcriptome analysis in response to GA3 was performed during the growth and development of ‘Meizao’ cherry during two critical periods of development. In total, 765 and 186 differentially expressed genes were identified, which were then compared to determine their biological functions based on GO and metabolic pathway enrichment. In this study, GA3 treatment was carried out at two key stages of sweet cherry development: anthesis and the hard-core stage. A one-to-one comparison of parthenocarpic fruit development after GA3 treatment was conducted, and differences were compared. The findings might result in a relatively comprehensive follow-up analysis to identify important genes linked to sweet cherry fruit production and that participate in functional pathways. Furthermore, it will be helpful to explore the potential mechanisms regulating and controlling the production of sweet cherry fruit.

### The effect of gibberellin on gene expression in the development of parthenogenetic sweet cherry

**Fig. 11:**
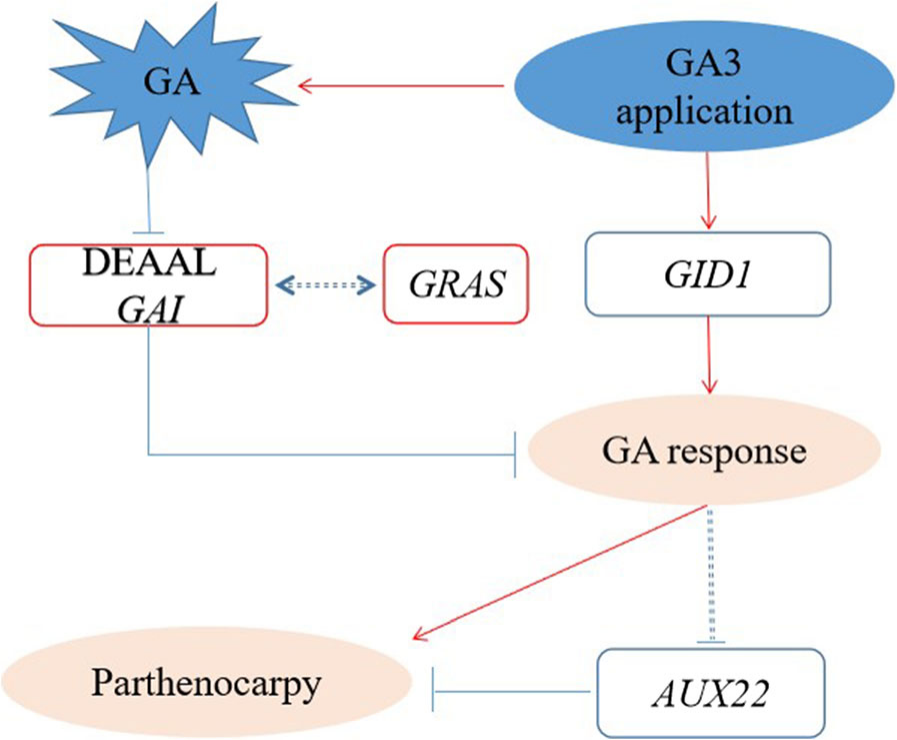
A model for parthenocarpy molecular regulation in sweet cherry. The red and blue frames show the up-regulated and down-regulated genes, respectively. The red and green lines indicate positive and negative regulation, respectively. The dashed line shows hypothetical interactions

In agriculture, the production of parthenocarpic seedless grapes often involves treating flower buds with gibberellin before flowering [32]. After GA3 application, the response regulates expression of *DELLA* and *GID1* via feedback. GA application decreases expression of auxin-responsive proteins, leading to parthenocarpy. Moreover, other factors appear to have a regulatory role in parthenocarpy; for example, *GRAS* was upregulated after GA3 application (Fig. 11). Therefore, analysis of hormone-related gene expression during the development of sweet cherry in two stages can help to further determine the influence of gene expression differences on sweet cherry parthenocarpy and elucidate the mechanism of parthenocarpy. Previous studies have shown that *SAUR* can promote cellular expansion in *Arabidopsis* [33, 34], and *SAUR* was upregulated in the first stage in the IAA signal transduction pathway, which is consistent with the results shown in Fig. 2. Expression of DEGs induced by GA3 is shown in Additional file 2. Differentially expressed genes associated with auxin synthesis and the auxin inhibitor IAA also showed patterns of upregulated expression at the first stage. Accordingly, these auxin-related differential gene expression patterns may be a manifestation of auxin balance feedback adjustment. The *GH3* gene maintains the dynamic balance of auxin in plants by encoding IAA-amido synthase, which converts active IAA into a bound form [7, 35]. In the hard-core stage of sweet cherry, expression of the *GH3* gene and the auxin inhibitor *AUX/IAA* was downregulated, further explaining the auxin feedback regulation. Different hormones can play cooperative roles through signaling, and one hormone that acts synergistically with auxin is BR. Indeed, studies have shown that auxin promotes plant growth by synthesizing BR [36].

In the BR response pathway, upregulated expression of the receptor gene *BRI1* indicates an association with the GA-induced BR response. In this study, genes related to BR synthesis displayed upregulated expression in the two critical stages of sweet cherry development, which is consistent with the synergistic effect of auxin and brassinolide. The study of Fu showed that external application of BR can stimulate cell division in cucumber and lead to parthenocarpy [37]. It is speculated that expression of genes related to BR synthesis and signal transduction in sweet cherry during seed development after gibberellin treatment is upregulated relative to that in untreated sweet cherry. GA may also promote the parthenogenesis of sweet cherry through similar mechanisms.

It has been reported that a high level of ethylene can cause parthenogenesis in tomato [38]. The results of our study showed that in the first period, expression of genes related to ethylene synthesis and signal transduction was upregulated after GA3 treatment. Abscisic acid also participates in the development of the seed coat in *Arabidopsis* and regulates cell wall metabolism by reducing cell wall PG, PE and XET [39, 40]. In the first critical period of development of sweet cherry, expression of a gene related to seed growth was detected by analyzing seed development-related gene patterns, consistent with the upregulated expression of the abscisic acid receptor and genes related to transmembrane transport at the two critical periods. However, no genes associated with seed coat development, which may only be expressed a short time after treatment, were detected in the hard-core period. In summary, genes associated with GA, auxin and BR were significantly upregulated after GA3 treatment, which may be associated with parthenogenesis. Regardless, the specific mechanism needs further study.

### Gibberellin promotes cell wall relaxation and induces fruit enlargement

The cell wall is composed of cellulose microfilaments embedded in the hemicellulose stromal layer through noncovalent bonding [41]. In the process of fruit enlargement, expansion proteins promote noncovalent dissociation, and glucanase and xylanase loosen the hemicellulose matrix [16]. Expression of DEGs associated with cell division after GA3 treatment is presented in Additional file 3. According to our results, glucanase gene ncRNA was significantly upregulated in the first stage after GA3 treatment, and the endoglucanase gene *ENDO17* was upregulated in the second stage. The two expansion genes *EXPA2* and *EXPB1* were significantly upregulated in the anthesis period; however, only one gene, *EXPA10*, was detected in the second period, and its expression was downregulated. Genes encoding glucanase, xylanase and expansion were significantly upregulated in the first period after GA3 treatment, and one expansion gene was downregulated in the second period, suggesting that these genes play an important role in the process of sweet cherry enlargement. Previous studies in grape have found that the *XET* gene was significantly upregulated during the first rapid growth stage and then decreased over time [42, 43], which is not consistent with the results. The main reason may be that different species exhibit different expression patterns. The growth and developmental period of sweet cherry is short. However, after GA3 treatment, the rapid growth period of sweet cherry was relatively long, and glucanase, xylanase and expansion genes were highly expressed. It is known cell wall modification plays a key role in exogenous GA3-induced fruit enlargement. In addition to genes associated with cell wall relaxation, cytoskeleton modification genes also play an important role in the process of GA3-induced fruit enlargement. According to the analysis of protein groups in the second and third period of grape development, expression of 3 actin and 10 tubulin genes was upregulated.

### Gibberellin-induced fruit setting in sweet cherry

Pollination is a key step in the development of flowers to fruits. Egg cells develop into seeds after fertilization by pollen, and the developing zygotes stimulate the ovary to form a fruit. We identified three genes that may play an important role in sweet cherry fruit set. First, the effects of ethylene include promotion of fruit ripening, and ethephon is used as a thinning agent for many fruit trees [44–46]. AP2/ERF is one of the largest transcription factor families in plants [47], the members of which are mainly involved in the regulation of such processes as plant growth [48, 49], flower development [50], fruit development [51] and seed development [52]. In the present study, six AP2/ERF transcription factors showed altered expression in the flowering stage, and four of them are ethylene-responsive transcription factors. We speculate that these genes may play an important role in the transition from flowers to fruit, thus promoting fruit set. Altered expression of three AP2/ERF transcription factors was found in the hard-core stage, possibly regulating ovule senescence via changes in the expression patterns of different genes. In addition, the ethylene synthesis pathway enzymes 1-aminocyclopropane-1-carboxylate oxidase (*ACO*) and 1-aminocyclopropane-1-carboxylate synthase (*ACS*) were upregulated after GA treatment. These enzymes may act by altering GA signaling to promote the occurrence of parthenocarpy and fruit set. Second, the *YUCCA* gene plays an important role in the biosynthesis of auxin and plant development [53]. Expression of DEGs associated with fruit set after GA3 treatment are listed in Additional file 4. The *YUCCA10* gene encoding indole-3-pyruvate monooxygenase was significantly upregulated after GA treatment. The *YUCCA* gene plays an important role in vegetative growth and reproductive development of plants and regulates the formation of the floral meristem and the development of lateral organs [54]. Some studies have reported that the content of auxin in the ovary increases after GA treatment [55, 56]. Overall, GA may play a role in auxin accumulation in the ovary and in turn act as a signal to affect fruit set. Third, the gene coding for ABA 8′-hydroxylase was upregulated after GA treatment in the second stage, and this gene is thought to be the main reason for the decrease in ABA [57]. A high ABA content causes fruit to drop. After GA treatment, expression of this gene was upregulated, which was beneficial to the fruit. These results are consistent with the results shown in Fig. 2, indicating that this period is critical for fruit set. These results indicate that auxin, ethylene and abscisic acid may regulate fruit set in sweet cherry after GA treatment.

## Conclusions

To the best of our knowledge, this study is the first to provide a dynamic analysis of differences in differentially expressed genes related to parthenogenesis, fruit enlargement and fruit set in sweet cherry fruit due to GA3 treatment. We selected two critical periods during the development of sweet cherry, and genes involved in many metabolic processes changed significantly after a week of GA treatment, including hormone-related genes, transcription factors and cell division genes. Further analysis of these genes will provide a new approach for studying fruit set in sweet cherry. Additionally, our research results provide a relatively complete molecular platform for future research on mechanisms of parthenocarpy, fruit enlargement and fruit set in sweet cherry.

## Methods

### Plant material

Trees of the ‘Meizao’ variety of sweet cherry (*Cerasus pseudocerasus* G. Don Meizao) were treated in the anthesis and hard-core stages at the commercial greenhouse in Jueyu (Tai’an, China) in 2017. All plant materials used in our study are regular species, which do not require any permissions. Experimental materials were obtained from three 10-year-old trees growing under similar conditions. We selected four major branches with similar growth status and direction per tree and divided them into two groups: one group was used for fruit set statistics and the other group for sampling. Every group included two branches, one for GA3 (300 mg/L of GA3 containing 0.1% (v/v) Tween 20) treatment and another as a control (growing under the natural state). After treatment with GA3, 30 cm*25 cm transparent paper bags were applied to cover flowers to avoid interference from pollen and to allow for normal management. Blossoming to maturity was divided into five periods: anthesis, the first enlargement period, the hard-core period, the second enlargement period and the mature period, respectively. Samples were collected from the two important stages for parthenogenetic fruit (anthesis and hard-core stages); ten fruits were collected from each branch at the anthesis (7 days after treatment, 7 DAT) and hard-core (27 DAT) stages and used for transcriptome analysis, with three branches of three trees used as three biological replicates. A workflow chart of the analytical process is shown in Additional file 5.

### RNA extraction

The two key developmental periods of sweet cherry, anthesis and hard-core were examined. In each stage, RNA was extracted from sweet cherries treated or not with GA3. Total RNA was extracted using the RNAprep Pure TIANGEN kit (TIANGEN, DP441, Beijing). The quality and quantity of the RNA were determined using a Kaiao K5500 spectrophotometer (Kaiao, Beijing) followed by 1% agarose gel electrophoresis (18S and 28S bands). The integrity and concentration of the RNA samples were detected using Agilent 2100 RNA Nano 6000 Assay Kit (Agilent Technologies, CA, USA), and high-quality RNA was sent to Beijing Genomics for further quality assessment.

### Library preparation for transcriptome analysis

After evaluating the total RNA samples, mRNA was enriched using magnetic beads containing oligo (dT) primers. Fragmentation buffer was added to the obtained mRNA to produce short fragments, which were then used as templates. First-strand cDNA was synthesized using six-base random primers; buffer, dNTPs, RNaseH and DNA polymerase I were added to synthesize the second strand. The cDNA obtained was purified using a QIAquick PCR kit and eluted with EB buffer. The purified double-stranded cDNA was subjected to end repair, base A addition and sequencing, and fragment sizes were then determined by agarose gel electrophoresis and PCR amplification. The constructed library was sequenced using the Illumina platform.

### Illumina read processing and de novo assembly

Illumina high-throughput sequencing results were originally obtained as raw image data files. After CASSAVABase base recognition (base calling), the data were transformed into the original sequence (sequenced reads), and the results were stored in FASTQ (fq) file format. In this format, the ASCII code value corresponding to each base quality character minus 33 (Sanger quality system) is the sequencing quality score of the base. Different scores represent different nucleotide sequencing error rates, such as score values of 20 and 30, indicating base sequence error rates of 1 and 0.1%, respectively. To ensure the quality of the data for analysis, we filtered out sequencing linkers, low-quality sequences and reads with an N ratio greater than 5%. After filtering, the total number of bases with a mass value greater than 30 (error rate less than 0.1%) was divided by the total number of bases to obtain the Q30 value; the higher this number, the higher is the sequencing quality. A follow-up analysis based on the clean reads was performed.

### Functional annotation and classification

High-quality clean reads were obtained by removing sequences of sequencing joints, low-quality sequences (Q is less than 19 bases), and reads with an N ratio of more than 5%. TopHat software [20] with Bowtie 2 [58] were employed for alignment of the sequences to the European sweet cherry genome located at https://www.rosaceae.org/species/prunus_avium/genome_v1.0.a1.

No more than two base mismatches were allowed, and annotation results for genes were obtained by comparing them with the European sweet cherry genome data. In addition, NCBI, UniProt, GO and KEGG were used for annotations. For GO analysis, the NCBI NR database was first used for BLAST alignment, and Blast2 GO was utilized to obtain GO entries corresponding to each gene. Enrichment analysis was performed for each pathway in KEGG to determine the significance of differentially expressed genes (enrichment of pathway).

### DEG analysis

The clean reads from the two developmental stages were obtained through transcriptome sequencing as described above, and the complete dataset excluded sequences with more than two base mismatches [59]. The amount of mRNA produced was calculated using FPKM [60] to estimate gene expression levels, which were compared between the two developmental stages using DESeq. Genes with |log2Ratio| ≥ 1 and q < 0.05 were selected as significant differentially expressed genes [61]. These differentially expressed genes were subjected to GO analysis and KEGG metabolic pathway enrichment analysis based on the calculation of the number of genes for each item and hypergeometric test results after *p*-value correction with a threshold of q < 0.05. The GO items satisfying this condition were defined as significantly enriched among the differentially expressed genes. A pathway defined by a Q-value ≤0.05 (corrected p-value) is a significantly enriched KEGG pathway for differentially expressed genes, and the definition of a GO item meeting the condition for significant enrichment among differently expressed genes is a Q-value ≤0.05 (pathway corrected p-value) based on the difference in gene expression enrichment in a KEGG pathway.

### Quantitative real-time PCR (qRT-PCR) validation

To verify the results of the transcriptome analysis, we selected genes involved in fruit set, cell division and parthenocarpy. Total RNA was reverse transcribed (1 μg per sample) in a 20-μl reaction using a cDNA reverse transcription synthesis kit (RR047A, TaKaRa, China); the primers used are listed in Additional file 1. The SYBR® Premix Ex Taq TM (Tli RNaseH Plus) kit (Thermo Fisher) was employed for the fluorescence quantitative PCR reaction. The reaction system included the following: 12.5 μL SYBR® Premix Ex Taq (2 ×), 1 μL each forward and reverse primers, 1 μL cDNA, and ddH_2_O to 25 μL. The experimental design included 3 technical replicates. The fluorescence quantitative PCR reaction conditions were as follows: 35 ~ 40 cycles of pre-denaturation at 95 °C for 30 s, denaturation at 95 °C for 5 s, and annealing at 60 °C for 30 s. After the reaction, a fluorescence curve and a melting curve were obtained, and the comparative Ct (2-ΔΔCt) method was used for data analysis [62].

## Additional file

Additional file 1: The primers used in this article. (DOC 78 kb)

Additional file 2: Expression of DEGs induced parthenocarpy by GA3. (DOC 34 kb)

Additional file 3: Expression of DEGs associated with cell division after GA3 treatment. (DOC 20 kb)

Additional file 4: Expression of DEGs associated with fruit setting after GA3 treatment. (DOC 22 kb)

Additional file 5: the workflow chart of the analytical process. (JPG 105 kb)

## Data Availability

The dataset supporting the conclusions of this article is available in the GenBank data libraries with the accession number PRJNA529517 (https://www.ncbi.nlm.nih.gov/bioproject/PRJNA529517).

## Abbreviations

DAT: Days after treatment
DEG: Differentially genes expressed
GO: Gene Ontology
KEGG: Kyoto Encyclopedia of Genes and Genomes
qRT-PCR: Quantitative real-time PCR
TF: transcription factors
