# Supplementary material for: Identification and characterization of cherry (Cerasus pseudocerasus G. Don) genes responding to parthenocarpy induced by GA3 through transcriptome analysis
Source: BMC Genet. 2019 Aug 1;20:65. doi: 10.1186/s12863-019-0746-8 (PMC6670208; doi:10.1186/s12863-019-0746-8)
Supplement: Supplementary file 1 — The primers used in this article. (DOC 78 kb) [file 12863_2019_746_MOESM1_ESM.doc]

Additional file 1

The primers involved in this article

| Primes name | Primes squence |
| --- | --- |
| PavACTIN(qRT)-F | AATGGTGAAGGCTGGATTTG |
| PavACTIN(qRT)-R | ACATCCTTCTGACCCATAC |
| Pav_co4015479.1_g010.1.mk-F | AAGTATCCAACCATTGATGT |
| Pav_co4015479.1_g010.1.mk-R | CCTCTTATGCTGCTGATT |
| Pav_co4016743.1_g010.1.br-F | GCCACATTACTTAGCAATCA |
| Pav_co4016743.1_g010.1.br-R | TTGAAGAACCGACCTGAG |
| Pav_co4073645.1_g010.1.mk-F | GAGCGAGCACTTCTTGAG |
| Pav_co4073645.1_g010.1.mk-R | CATTCACCATCCACTTCCAT |
| Pav_sc0000026.1_g140.1.mk-F | TATGGCAATGCTGTAGAA |
| Pav_sc0000026.1_g140.1.mk-R | CACTTCACCTGATAACAAG |
| Pav_sc0000030.1_g1280.1.mk-F | CCAACATTCTCTACACATT |
| Pav_sc0000030.1_g1280.1.mk-R | AGTTAAGGTCAGCACATA |
| Pav_sc0000129.1_g1090.1.mk-F | TTGTTGTTGCCTCATCTC |
| Pav_sc0000129.1_g1090.1.mk-R | ACATTGTGGTAGTCATAAGTG |
| Pav_sc0000138.1_g830.1.br-F | AACACCACCACCTCAATT |
| Pav_sc0000138.1_g830.1.br-R | AATGTCTTTGGGCTCCTT |
| Pav_sc0000195.1_g560.1.mk-F | CAATCACAGGGAACAGTAA |
| Pav_sc0000195.1_g560.1.mk-R | GCTCCATCTCCAATCATT |
| Pav_sc0000244.1_g040.1.mk-F | GCCACATTACTTAGCAATCAA |
| Pav_sc0000244.1_g040.1.mk-R | AACCAACCTGAGCAACAT |
| Pav_sc0000464.1_g350.1.mk-F | ATGAAGCCTAAGATTGTG |
| Pav_sc0000464.1_g350.1.mk-R | CAAGTTGGAGTAATAATGC |
| Pav_sc0000467.1_g830.1.br-F | GTGCCACTGACAAGAAGAA |
| Pav_sc0000467.1_g830.1.br-R | TCAGGACCTTGAAGATGTAGAT |
| Pav_sc0000556.1_g030.1.mk-F | TTATCAACGGCTCAGATT |
| Pav_sc0000556.1_g030.1.mk-R | TTTAGATGCGATGGAAATG |
| Pav_sc0000558.1_g910.1.mk-F | CTCTGCTTGAATCGTATG |
| Pav_sc0000558.1_g910.1.mk-R | CTGTATCTTAATTGCTTGTTG |
| Pav_sc0000600.1_g680.1.mk-F | CTACACATTACTCAACTG |
| Pav_sc0000600.1_g680.1.mk-R | TACCTAACAACCTGAATA |
| Pav_sc0000713.1_g640.1.mk-F | ATTACAAGGAGTCAGGTTCA |
| Pav_sc0000713.1_g640.1.mk-R | AAGGATGCTGGTTGCTAA |
| Pav_sc0000714.1_g390.1.br-F | AATGAGGTTCCAGGCATA |
| Pav_sc0000714.1_g390.1.br-R | TTATCCCAGCATCAACAAG |
| Pav_sc0000780.1_g280.1.br-F | CCAAGAGGCATAGGAAGGT |
| Pav_sc0000780.1_g280.1.br-R | TACGAGCAAGACGACGAAT |
| Pav_sc0000848.1_g080.1.mk-F | AAGCAGAAGTTGAGGTATT |
| Pav_sc0000848.1_g080.1.mk-R | CGGTCTTGAATTGTAACG |
| Pav_sc0000848.1_g330.1.mk-F | AAGTATCCAACCATTGATGT |
| Pav_sc0000848.1_g330.1.mk-R | CCTCTTATGCTGCTGATT |
| Pav_sc0000862.1_g450.1.br-F | TCTACGAGGAGACCAGAG |
| Pav_sc0000862.1_g450.1.br-R | CGTGCTCAGTGTAAGTCA |
| Pav_sc0000886.1_g690.1.mk-F | CAACCAACAGCAACTCTT |
| Pav_sc0000886.1_g690.1.mk-R | TCACTATCATAAGCCTTCCA |
| Pav_sc0001243.1_g200.1.mk-F | ATTGTCTTGCTTGTAGTA |
| Pav_sc0001243.1_g200.1.mk-R | CACCTATATCTGTTCCTT |
| Pav_sc0002234.1_g030.1.mk-F | ATTGAAGCATTAGCCATT |
| Pav_sc0002234.1_g030.1.mk-R | TCTCTATAAGTGTCATCCA |
| Pav_sc0002445.1_g100.1.mk-F | CCTCTATTGGTTGGTATG |
| Pav_sc0002445.1_g100.1.mk-R | TGCCGTCTTATTATTGTT |
| Pav_sc0003033.1_g190.1.mk-F | ATCACCGAGCAAGAATCA |
| Pav_sc0003033.1_g190.1.mk-R | TGTAGACTCCAAGCAATCA |
| Pav_sc0003135.1_g610.1.mk-F | GTATTGGAATTGATGGTTGATG |
| Pav_sc0003135.1_g610.1.mk-R | AGTCGCTCTTATCCTCTC |
| Pav_sc0005746.1_g060.1.mk-F | TGACTATGGAGGAGGATG |
| Pav_sc0005746.1_g060.1.mk-R | CATTGAGGTAAGAGTTGCT |
| Pav_sc0006281.1_g040.1.br-F | AGAAGATGAAGATGAAGCCG |
| Pav_sc0006281.1_g040.1.br-R | TGTTGTTGACCTGGTGGAT |
| Pav_sc0002136.1_g280.1.mk-F | AACAGGAATCGGCAAAGC |
| Pav_sc0002136.1_g280.1.mk-R | TGCTCCACCCTCCATCATA |
| Pav_sc0000257.1_g1300.1.mk-F | GAATCTGAGGTGGCAGTTATCA |
| Pav_sc0000257.1_g1300.1.mk-R | CCTTAGTTTCAATCACACGGC |
