# Supplementary material for: Identification and characterization of cherry (Cerasus pseudocerasus G. Don) genes responding to parthenocarpy induced by GA3 through transcriptome analysis
Source: BMC Genet. 2019 Aug 1;20:65. doi: 10.1186/s12863-019-0746-8 (PMC6670208; doi:10.1186/s12863-019-0746-8)
Supplement: Supplementary file 2 — Expression of DEGs induced parthenocarpy by GA3. (DOC 34 kb) [file 12863_2019_746_MOESM2_ESM.doc]

Additional file 2

Expression of DEGs induced parthenocarpy by GA3

| Genes | Log2 Fold Change | Gene annotation |
| --- | --- | --- |
| T1C1 |  |  |
| Pav_sc0000095.1_g1110.1.mk | 5.45724796998513 | GA2ox |
| Pav_sc0000138.1_g830.1.br | 4.44304139521031 | SAUR32 |
| Pav_co4073645.1_g010.1.mk | 1.98037099237683 | SCL1 |
| Pav_sc0000195.1_g560.1.mk | 2.3939037236711 | ILR1 |
| Pav_sc0000848.1_g330.1.mk | 3.09869360135758 | YUCCA |
| Pav_sc0003033.1_g190.1.mk | –3.55224132857802 | SCL3 |
| Pav_sc0000129.1_g1090.1.mk | 1.68068017726551 | P450 |
| Pav_sc0000713.1_g640.1.mk | 1.54392035732455 | BEL1 |
| T2C2 |  |  |
| Pav_sc0000716.1_g200.1.mk | –1.60274199390278 | AUX22 |
| Pav_sc0002234.1_g030.1.mk | 1.9897694654479 | CYCPA3 |
| Pav_sc0000464.1_g350.1.mk | 1.11067656965906 | DELLA |
| Pav_sc0000030.1_g1280.1.mk | 2.57471211290008 | AUX3 |
| Pav_sc0000848.1_g080.1.mk | 1.52814176348772 | GID1B |
| Pav_sc0003135.1_g610.1.mk | 3.27975040780843 | GA2ox |
