# Supplementary material for: Identification and characterization of cherry (Cerasus pseudocerasus G. Don) genes responding to parthenocarpy induced by GA3 through transcriptome analysis
Source: BMC Genet. 2019 Aug 1;20:65. doi: 10.1186/s12863-019-0746-8 (PMC6670208; doi:10.1186/s12863-019-0746-8)
Supplement: Supplementary file 3 — Expression of DEGs associated with cell division after GA3 treatment. (DOC 20 kb) [file 12863_2019_746_MOESM3_ESM.doc]

Additional file 3

Expression of DEGs associated with cell division after GA3 treatment

| Genes | Log2 Fold Change | Gene annotation |
| --- | --- | --- |
| T1C1 |  |  |
| Pav_sc0000780.1_g280.1.br | –2.15156499084565 | H4 |
| Pav_sc0000600.1_g680.1.mk | –2.92597114948488 | CDK B2–2 |
| Pav_sc0000556.1_g030.1.mk | –3.27691375000291 | H3.2 |
| Pav_sc0000558.1_g910.1.mk | –3.57620925472513 | CDC45 |
| Pav_sc0000862.1_g450.1.br | –3.48822239012501 | H4 |
| Pav_sc0002445.1_g100.1.mk | 3.89996519603023 | Cell cycle protein |
| Pav_sc0000467.1_g830.1.br | –2.35867586168819 | H2B |
