# Supplementary material for: Identification and characterization of cherry (Cerasus pseudocerasus G. Don) genes responding to parthenocarpy induced by GA3 through transcriptome analysis
Source: BMC Genet. 2019 Aug 1;20:65. doi: 10.1186/s12863-019-0746-8 (PMC6670208; doi:10.1186/s12863-019-0746-8)
Supplement: Supplementary file 4 — Expression of DEGs associated with fruit setting after GA3 treatment. (DOC 22 kb) [file 12863_2019_746_MOESM4_ESM.doc]

Additional file 4

Expression of DEGs associated with fruit setting after GA3 treatment

| Genes | Log2 Fold Change | Gene annotation |
| --- | --- | --- |
| T1C1 |  |  |
| Pav_co4015479.1_g010.1.mk | 5.873025445 | YUCCA |
| Pav_sc0000848.1_g330.1.mk | 1.63166010837502 | YUCCA10 |
| Pav_sc0000886.1_g690.1.mk | 4.7162027601994 | ACS |
| Pav_sc0000714.1_g390.1.br | 5.15732809007253 | ACO |
| T2C2 |  |  |
| Pav_sc0002234.1_g030.1.mk | 1.9897694654479 | CYP707A3 |
| Pav_sc0006281.1_g040.1.br | 3.94884174715176 | AGL103 |
| Pav_sc0002136.1_g280.1.mk | –2.10686403519515 | AGL11 |
| Pav_sc0000257.1_g1300.1.mk | 1.89919421680317 | AGL24 |
