# Supplementary figures and images for: Identification and characterization of cherry (Cerasus pseudocerasus G. Don) genes responding to parthenocarpy induced by GA3 through transcriptome analysis
Source: BMC Genet. 2019 Aug 1;20:65. doi: 10.1186/s12863-019-0746-8 (PMC6670208; doi:10.1186/s12863-019-0746-8)

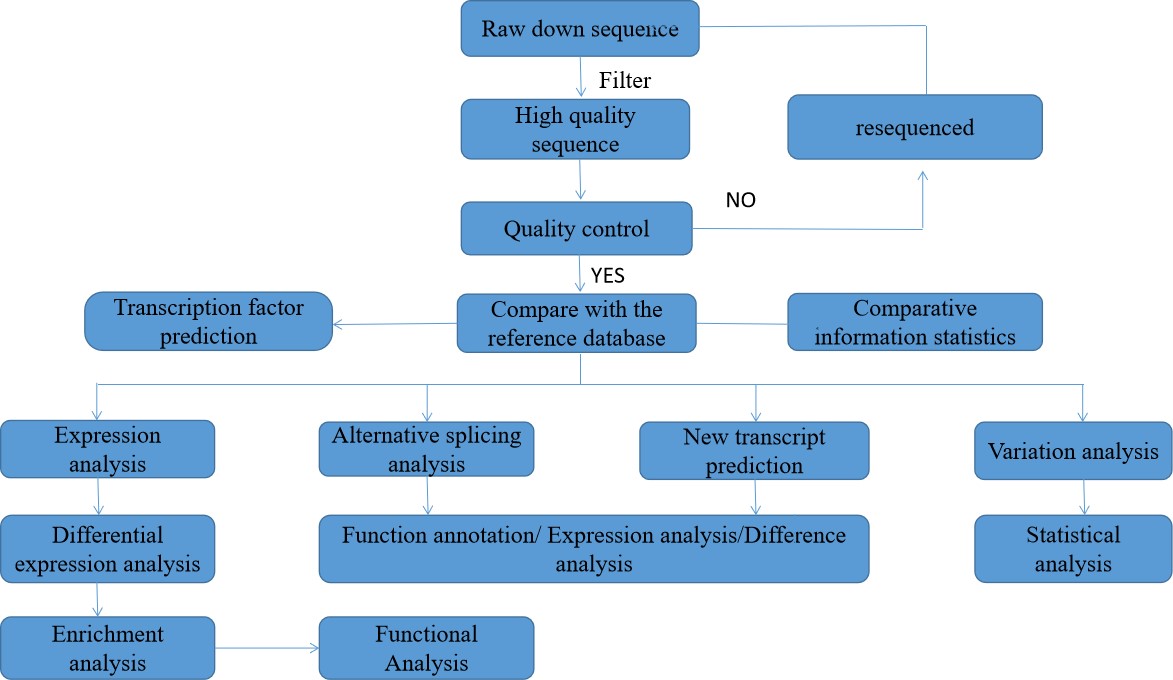

Supplement: Supplementary file 5 — the workflow chart of the analytical process. (JPG 105 kb) [file 12863_2019_746_MOESM5_ESM.jpg]
